# The combination of cyclin D1 and MRI radiomics parameters improves breast cancer diagnosis and NACT efficacy assessment

**DOI:** 10.5937/jomb0-60127

**Published:** 2026-02-27

**Authors:** Yufei Fu, Yizhi Shi, Zhen Wang, Xin Zhou

**Affiliations:** 1 Huangshi Central Hospital, Affiliated Hospital of Hubei Polytechnic University, Edong Healthcare Group, Department of Radiology, Hubei, China

**Keywords:** cyclin D1, MRI, radiomics, breast cancer, neoadjuvant chemotherapy, ciklin D1, magnetna rezonanca, radiomika, rak dojke, neoadjuvantna hemoterapija

## Abstract

**Background:**

The purpose of this study is to evaluate the combined diagnostic performance of Cyclin D1 and magnetic resonance imaging (MRI) parameters in breast cancer (BC), while also assessing their ability to forecast treatment response to neoadjuvant chemotherapy (NACT), so as to provide evidence for individualized treatment strategies.

**Methods:**

A total of 154 BC patients and 148 healthy women matched with BC patients in age and BMI were enrolled in this study. Peripheral blood Cyclin D1 quantification utilized enzyme-linked immunosorbent assay (ELISA), and CA15-3 and CA27.29 (both tumor markers) measurements were made using automated electrochemiluminescence immunoassay. Magnetic resonance imaging (MRI) was conducted to obtain dynamic contrast-enhanced scans, from which radiomics parameters (PSIER, TSICS, ADC) were derived. Diagnostic accuracy for BC and predictive value for chemotherapy response were evaluated through correlation analysis (Pearson), receiver operating characteristic (ROC) curves, and multivariate regression modeling.

**Results:**

Cyclin D1 levels were elevated in BC patients compared to healthy controls and showed a positive connection with CA15-3 and CA27.29 (P&lt; 0.05). The diagnostic performance of Cyclin D1 alone yielded an AUC of 0.830, whereas a combined model incorporating MRI parameters (PSIER, TSICS, and ADC) significantly improved discrimination (AUC = 0.935, sensitivity 86.36%, specificity 87.84%). During NACT, Cyclin D1 levels declined dynamically. A predictive model integrating Cyclin D1 and imaging biomarkers achieved an AUC of 0.775 for identifying poor NACT responders (sensitivity 88.37%, specificity 56.76%).

**Conclusions:**

The combination of Cyclin D1 and MRI parameters enhances both BC diagnostic accuracy and NACT efficacy prediction.

## Abstract (sr)

**Uvod:**

Cilj ove studije je da se proceni kombinovana dijagnostička efikasnost parametara ciklina D1 i magnetne rezonance (MRI) kod raka dojke (RD), a istovremeno da se proceni njihova sposobnost predviđanja odgovora na lečenje neoadjuvantnom hemoterapijom (NAHT), kako bi se pružili dokazi za individualizovane strategije lečenja.

**Metode:**

U ovu studiju je uključeno ukupno 154 pacijenta sa rakom dojke i 148 zdravih žena, uparenih sa pacijentima sa rakom dojke po starosti i ITM. Kvantifikacija ciklina D1 u perifernoj krvi koristila je enzimski imunosorbentni test (ELISA), a merenja CA15-3 i CA27.29 (oba tumorska markera) izvršena su korišćenjem automatizovanog elektrohemiluminiscentnog imunotesta. Magnetna rezonanca (MRI) je sprovedena da bi se dobili dinamički snimci sa kontrastnim pojačanjem, iz kojih su izvedeni radiomički parametri (PSIER, TSICS, ADC). Dijagnostička tačnost za rak dojke i prediktivna vrednost za odgovor na hemoterapiju procenjene su pomoću korelacione analize (Pearson), ROC krivih i multivarijantnog regresionog modeliranja.

**Rezultati:**

Nivoi ciklina D1 bili su povišeni kod pacijenata sa rakom dojke u poređenju sa zdravim kontrolama i pokazali su pozitivnu vezu sa CA15-3 i CA27.29 (P &lt; 0,05). Dijagnostičke performanse samog ciklina D1 dale su AUC od 0,830, dok je kombinovani model koji uključuje MRI parametre (PSIER, TSICS i ADC) značajno poboljšao diskriminaciju (AUC= 0,935, osetljivost 86,36%, specifičnost 87,84%). Tokom NACT-a, nivoi ciklina D1 su dinamički opadali. Prediktivni model koji integriše ciklin D1 i bio-markere za snimanje postigao je AUC od 0,775 za identifikaciju osoba sa slabim NACT odgovorom (senzitivnost 88,37%, specifičnost 56,76%).

**Zaključak:**

Kombinacija parametara ciklina D1 i MRI poboljšava i dijagnostičku tačnost karcinoma bradavastog karcinoma i predviđanje efikasnosti NACT-a.

## Introduction

Breast cancer (BC) ranks among the most prevalent malignancies affecting women globally, with its incidence and mortality rates consistently high among female cancers, posing a significant threat to their health and well-being [1]. The ability to achieve early and accurate detection, coupled with tailored treatment plans, is pivotal for optimizing patient outcomes [2]. Contemporary diagnostic modalities for BC include imaging techniques like mammography, ultrasound, and magnetic resonance imaging (MRI). Although MRI excels in visualizing soft tissue structures and detecting abnormalities, its diagnostic utility is constrained by challenges such as a high likelihood of false-positive interpretations and limited efficacy in identifying microcalcifications [3]. Conventional biomarkers like Ki-67, estrogen receptor (ER), progesterone receptor (PR), and human epidermal growth factor receptor 2 (HER2) are integral to BC diagnosis and therapeutic decision-making. However, the invasiveness of tissue biopsies and tumor heterogeneity restrict the reliability of single-marker analyses in comprehensively reflecting the tumor's biological behaviour [4]. Radiomics analysis, as an emerging technology, has shown great potential in tumor characterization by leveraging large-scale feature extraction from medical images. Nonetheless, unimodal imaging approaches have inherent limitations, as they are susceptible to variations in image acquisition parameters and tumor heterogeneity [5].

Cyclin D1 is a pivotal regulator in the cell cycle network, facilitating G1-to-S phase progression through its binding to cyclin-dependent kinases 4 and 6 (CDK4/6) [6]. Studies have identified Cyclin D1 overexpression and gene amplification as common events in BC [7], with emerging evidence linking it to resistance against endocrine therapies [8]. However, its potential utility in BC has not been thoroughly investigated.

The central involvement of Cyclin D1 in BC oncogenesis suggests that its combination with MRI parameters could provide a more comprehensive tumor evaluation than single-parameter approaches. This multimodal strategy may not only enhance diagnostic accuracy but also enable dynamic monitoring of neoadjuvant chemotherapy (NACT) response through Cyclin D1 fluctuation patterns, allowing for adaptive treatment optimization and better prognostic outcomes.

## Materials and methods

### Research subjects

From June 2023 to October 2024, we prospectively recruited 154 BC patients and 148 healthy controls. Power analysis (G*Power 3.1) determined the sample size based on: two-tailed test, effect size=0.3, α err prob = 0.05, power=0.95 (minimum n = 134/group). Accounting for 10% potential attrition (no cases were ultimately shed), we targeted 2147 participants per group.

### Inclusion and exclusion criteria

Eligibility Criteria: BC patients: Age 18-75 with pathological BC confirmation; Planned for NACT at our center; Life expectancy 26 months; Complete medical records. Healthy controls: Age-matched health checkup participants; No previous major medical history; Normal physical examination results. Exclusion Criteria (all participants): Triple negative BC (ER/PR/HER2-negative); Other malignant tumors; Serious organ dysfunction such as heart, liver and kidney; MRI contraindications; Prior cancer therapies (radiotherapy, targeted therapy, or other chemotherapy; Pregnancy or lactation; Incomplete imaging or pathological data.

### Ethical statement

This study was conducted following the Helsinki Declaration and received approval from Huangshi Central Hospital's Institutional Review Board. Written informed consent was obtained from all participants prior to study enrollment. All collected data were anonymized to ensure patient confidentiality. Anonymization was achieved by removing all direct identifiers (name, ID number, contact information) and assigning unique study codes to participant records. The key linking codes to identifiers was stored securely and separately from the research data.

### Imaging inspection

MRI scans (GE Discovery 750w) were conducted on both breasts and underarm areas. The phase with maximal lesion enhancement in the dynamic series was analyzed. Standard clinical dynamic contrast-enhanced (DCE)-MRI protocols were employed, including T1 -weighted, T2-weighted, and DWI sequences. Key acquisition parameters were: Repetition time (TR) / Echo time (TE) = [~4.5/~1.7 ms for DCE, ~3000/~85 ms for t 2], slice thickness = [3 mm], field of view (FOV) = [340 x 340 mm], matrix size = [256x 256]. DCE-MRI was performed after intravenous injection of gadolinium-based contrast agent (0.1 mmol/kg body weight) with multiple time points acquired. Two radiologists interpreted the images per Breast Imaging Reporting and Data System (BI-RADS) standards, recording radiomics parameters, including peak signal intensity enhancement ratio (PSIER), time to signal intensity curve slope (TSICS), and apparent diffusion coefficient (ADC) values. Both radiologists were board-certified with over 5 years of experience in breast MRI interpretation and were blinded to the clinical and laboratory data.

### NACT

All BC patients underwent NACT following the doctor's advice after admission. For example, Luminal BC regimens commonly integrate anthracyclines and taxanes over 4-6 cycles, whereas HER2-positive cases are managed with trastuzumab plus pertuzumab therapy for 6-8 cycles. Post-treatment outcomes were assessed via modified Response Evaluation Criteria In Solid Tumors (mRECIST) [9], defining responses as complete response (CR), partial response (PR), stable disease (SD), or progressive disease (PD).

### Laboratory examination

Fasting venous blood was sampled from BC patients before (T0), during (T1), and after chemo - therapy (T2), with the serum separated by room-temperature standing (30 min) and subsequent centrifugation [T1 samples were collected at the midpoint of the planned NACT regimen (e.g., after 3 cycles for a 6-cycle regimen). T2 samples were collected within one week after the completion of the entire NACT course]. ELISA detection of serum Cyclin D1 (Kit purchased from Shanghai Enzyme Research Biotechnology Co., LTD., EK-BIO, MH41775): We added 100 mL of serum to coated plates and incubated them at 37°C for 90 min. Next, enzyme-labeled secondary antibody was dispensed (100 μL/well) to incubate at 37°C for 60 min. Color development was initiated using the kit-matched TMB substrate, and absorbance (OD) at 450 nm was measured immediately after reaction termination. A standard curve was plotted using standard concentrations (x-axis) versus corresponding OD values (y-axis). Serum separation was performed within 48 hours post-collection. Each assay batch included tri-level quality controls (QCs) at target concentrations of low (10 pg/mL), medium (100 pg/mL), and high (300 pg/mL) levels, processed alongside samples, with results required to fall within acceptable ranges (target ±2 SD).

In addition, we quantified pre-chemotherapy BC tumor markers (CA15-3 and CA27.29) via Cobas e602 electrochemiluminescence. Serum samples, including QCs, were loaded into the sample rack with parameters configured via instrument software. The automated process included sample dispensing, reagent addition, 18 min 37°C incubation, washing, magnetic separation, luminescent signal acquisition, data calculation, and final CA15-3/CA27.29 concentration output. Daily validation required two-tiered QC samples (CA15-3: low: 15 U/mL; high: 200 U/mL), with results evaluated per Westgard rules (1_3S_/2_2S_/R_4S_).

### Statistical methods

Statistical analysis was made with SPSS 24.0. (χ̄±s) was used to describe measurement data, whose comparisons employed the independent sample t test. Counting data [n(%)] was examined by the chi-square test. Correlations were analyzed by Pearson correlation coefficients. ROC curves were plotted for diagnostic value analysis, with the maximum Youden index employed for cut-off, sensitivity, and specificity determination. Statistical significance was indicated by P<0.05.

## Results

### Comparability of two groups of subjects

The statistical analysis of patients' age, family history, body mass index (BMI), and other baseline data revealed no notable inter-group differences (P>0.05), suggesting comparability (Table 1).

**Table 1:** Clinical baseline data of the two study groups.

Groups		Healthy controls	BC patients	*t* or *χ^2^*	*P*
n		148	154		
Age		56.67±6.15	57.25±9.10	0.651	0.516
Reproductive history	Yes/no	134/14	142/12	0.267	0.606
Menstrual status	Menopausal/<br>pre-menopausal	133/15	139/15	0.013	0.909
Family History of BC	Yes/no	13/135	18/136	0.691	0.406
BMI (kg/m^2^)		25.61±1.88	25.90±2.37	1.16	0.247
Luminal A/Luminal B/Her-2 was overexpressed		-	56/63/35		

### Clinical significance of Cyclin D1 in BC

**Figure 1:**
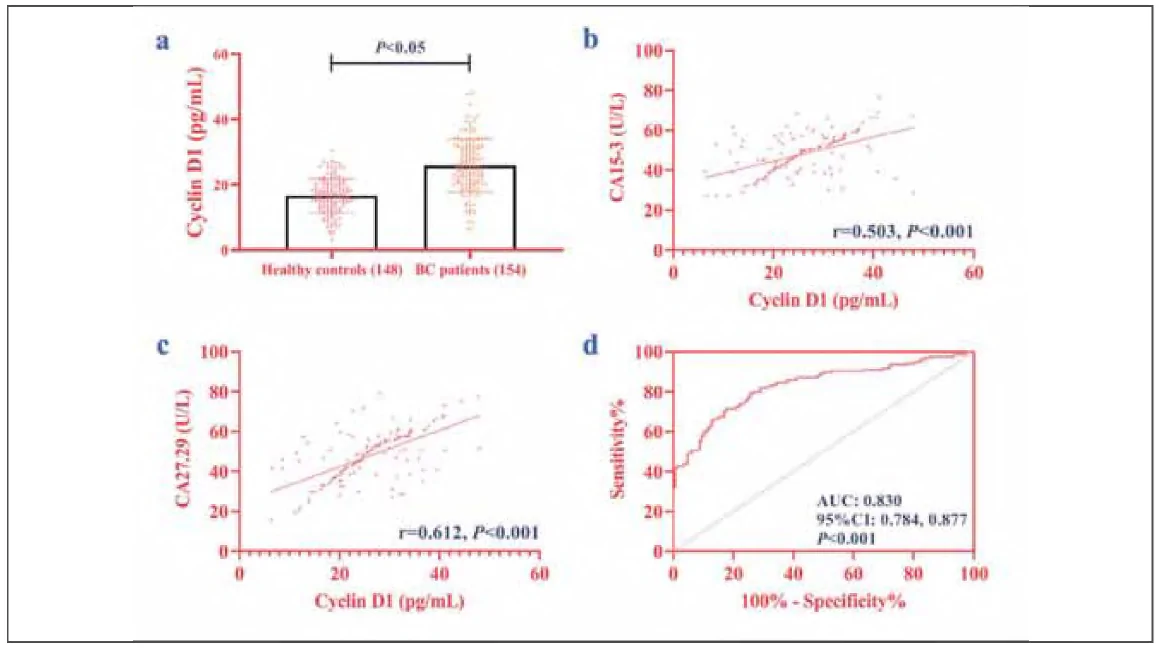
Diagnostic value of SLC7A11, p53, CD3^+^ T cells, and CD8^+^ T cells quantitation in EC. A: Comparison of SLC7A11, p53 mRNAs, CD3^+^ T cells, and CD8^+^ T cells between the two groups of subjects. B: ROC curves of SLC7A11, p53 mRNAs, CD3^+^ T cells, and CD8^+^ T cells for the diagnosis of EC. * indicates P < 0.05.

Cyclin D1 was markedly elevated in BC cases versus healthy controls (P<0.05). According to correlation analysis, Cyclin D1 had a strong and positive correlation with CA15-3 and CA27.29 in BC(P<0.05). ROC analysis demonstrated Cyclin D1's diagnostic potential for BC at a cutoff >21.38 pg/mL, yielding 71.43% sensitivity and 82.43% specificity (P<0.05, AUC=0.830) (Figure 1).

### Diagnostic effect of Cyclin D1 combined with MRI parameters on BC

**Figure 2:**
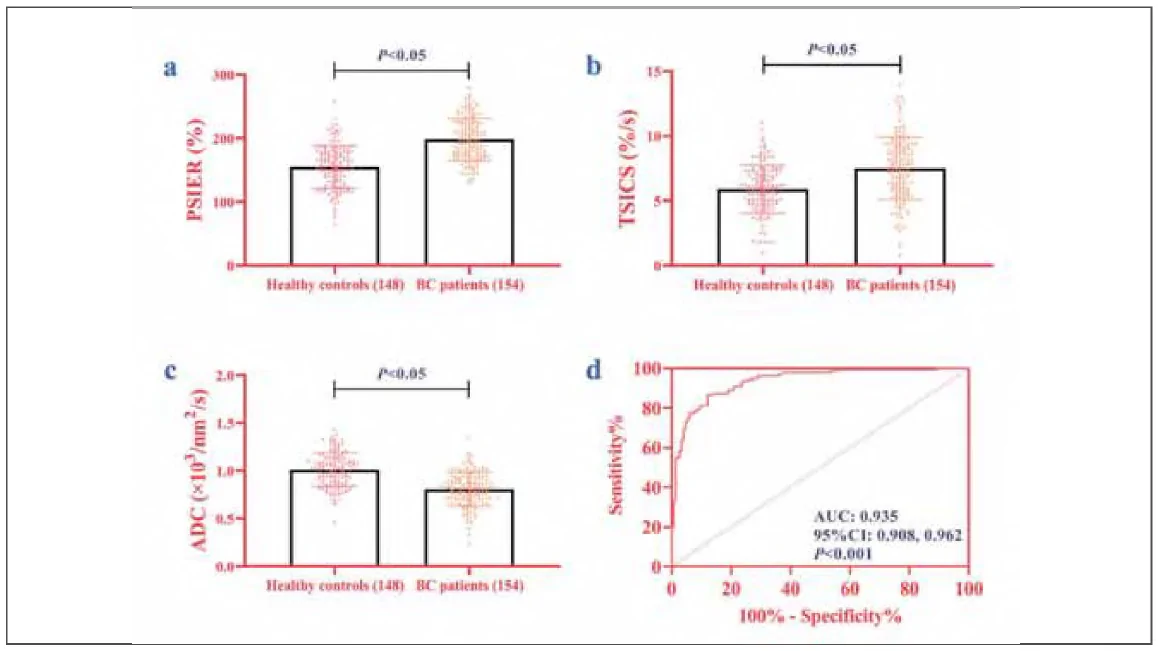
Analysis of the diagnostic efficacy of Cyclin D1 combined with MRI parameters for BC (T0). (a) Comparison of PSIER between BC patients and healthy controls. (b) Comparison of TSICS between BC patients and healthy controls. (c) Comparison of ADC between BC patients and healthy controls. (d) ROC curve of Cyclin D1 combined with MRI parameters for the diagnosis of BC. Note: baseline (T0).

Similarly, imaging findings demonstrated an elevation in PSIER and TSICS, as well as a decline in ADC, in BC patients compared to healthy controls (P<0.05). Regression analysis (healthy controls = 1, BC patients=2) identified PSIER, TSICS, and Cyclin D1 as independent risk factors for BC, whereas ADC exhibited a protective effect (P<0.05). Subsequently, we built a BC diagnostic model using baseline (T0) data by combining Cyclin D1 with MRI parameters based on regression coefficients (β) (Table 2). ROC analysis demonstrated that the combined model incorporating Cyclin D1 and MRI radiomics parameters significantly outperformed Cyclin D1 alone (AUC = 0.830) in diagnosing BC, achieving an excellent AUC of 0.935 (P<0.05) (Figure 2).

**Table 2:** Effect of Cyclin D1 and MRI parameters on BC (T0). Note: For logistic regression analysis of BC diagnosis, the dependent variable was coded as Healthy control = 0, BC patient = 1.

	β	S.E.	Wals	P	Exp (β)	95%CI
Cyclin D1	0.176	0.031	32.426	<0.001	1.035	1.123, 1.267
PSIER	0.034	0.006	32.190	<0.001	1.035	1.023, 1.047
TSICS	0.235	0.085	7.720	0.005	0.005	1.072, 1.494
ADC	-5.297	1.175	20.323	<0.001	1.193	0.001, 0.050
constant	-6.365	1.746	13.290	<0.001	-	-

### Dynamic changes in Cyclin D1 during NACT

**Figure 3:**
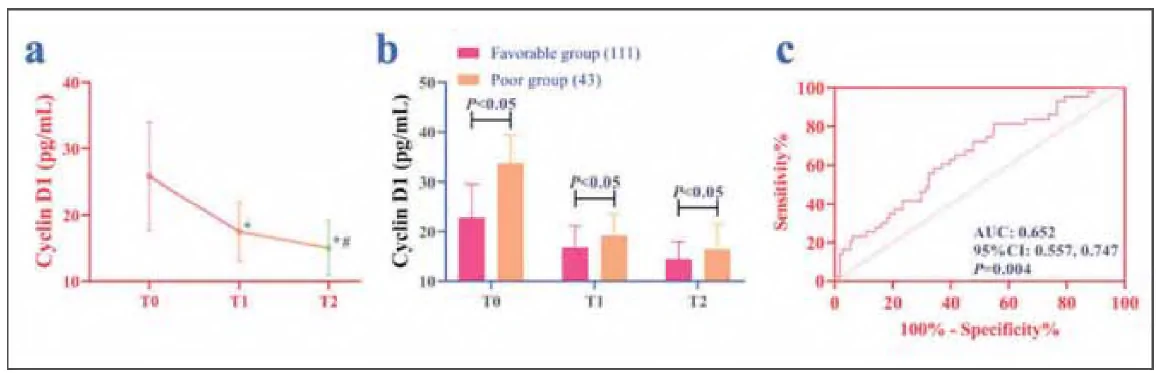
Relationship between Cyclin D1 and NACT efficacy in BC patients. (a) Dynamic changes of Cyclin D1 in T0-T1-T2, compared with T0 *P < 0.05, compared with T0 #P < 0.05. (b) Comparison of Cyclin D1 between favorable and poor groups (T0-T1-T2). (c) ROC curve of Cyclin D1 in the diagnosis of NACT response (T1). Note: baseline (T0), mid-treatment (T1), and post-treatment (T2).

Cyclin D1 expression in BC patients showed a progressive decrease from T0 to T2, with the lowest levels observed at T2 (P<0.05). Based on treatment outcomes, patients were categorized as favorable responses (CR+PR, n = 111) and poor responses (SD+PD, n=43). Cyclin D1 levels were found to be lower in the favorable-response group than in the poor-response group at all time points (T0, T1, T2; P<0.05). Further analysis revealed that a Cyclin D1 value >16.09 pg/mL at T1 predicted poor NACT efficacy with 81.40% sensitivity and 45.05% specificity (P<0.05, AUC=0.652) (Figure 3).

### Predictive performance of Cyclin D1 plus MRI parameters for NACT efficacy

**Figure 4:**
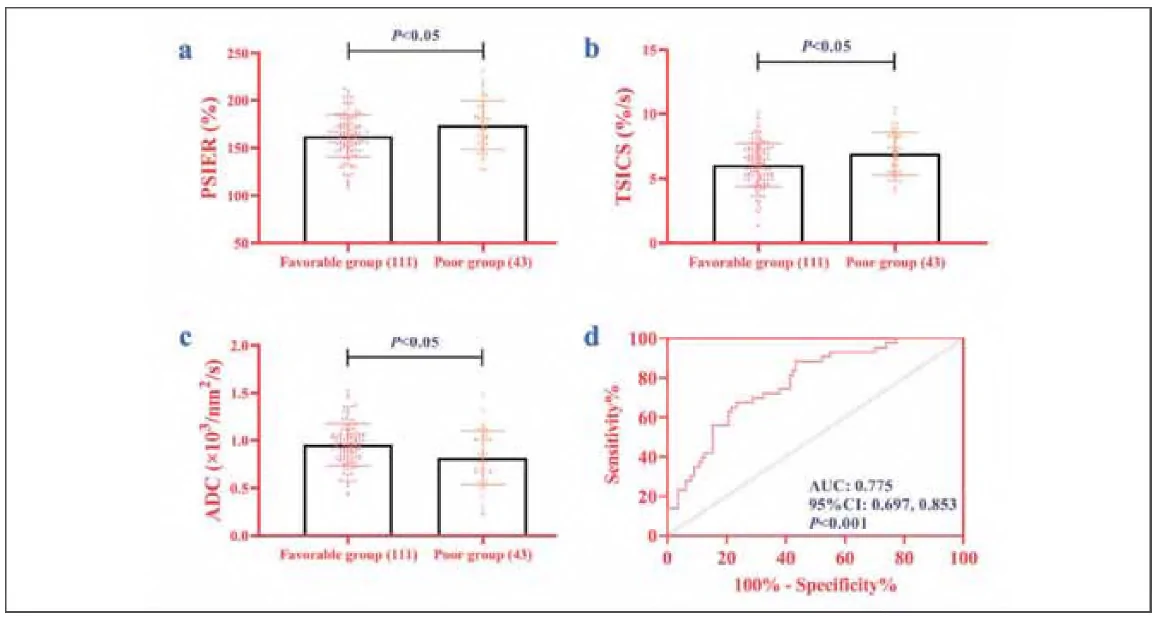
Diagnostic efficacy analysis of Cyclin D1 combined with MRI parameters for NACT (T1). (a) Comparison of PSIER between favorable group and poor group. (b) Comparison of TSICS between favorable group and poor group. (c) Comparison of ADC between favorable group and poor group. (d) ROC curve of Cyclin D1 combined with MRI parameters in the diagnosis of NACT response. Note: mid-treatment (T1).

We re-examined patients' images at T1 and found statistically lower PSIER and TSICS values in the favorable-response group compared to the poorresponse group, while ADC values were higher (P<0.05). Using treatment outcome (favorable=1, poor=2) as the dependent variable and Cyclin D1 levels plus parameters at T1 as independent variables, we derived a composite formula (Table 3). ROC analysis demonstrated that this formula predicted poor NACT response with 88.37% sensitivity and 56.76% specificity (P<0.05), it was also significantly better than Cyclin D1 alone (Au C=0.775) (Figure 4).

**Table 3:** Effect of Cyclin D1 and MRI parameters on the efficacy of NACT.

	β	S.E.	Wals	*P*	Exp (β)	95%CI
Cyclin D1	0.142	0.050	8.035	0.005	1.153	1.045, 1.272
PSIER	0.022	0.009	5.583	0.018	1.022	1.004, 1.041
TSICS	0.339	0.127	7.170	0.007	1.404	1.095, 1.799
ADC	-2.171	0.854	6.453	0.011	0.114	0.021, 0.609
constant	-7.478	2.282	10.738	0.001	-	-

## Discussion

This study presents the first investigation regarding the clinical utility of Cyclin D1 plus MRI parameters in BC diagnosis and NACT response prediction. The results showed up-regulated Cyclin D1 in BC patients and its positive correlation with tumor markers CA15-3 and CA27.29, highlighting its potential diagnostic value. In addition, the diagnostic model integrating Cyclin D1 with PSIER, TSICS, and ADC values demonstrated superior performance to individual parameters, achieving an AUC of 0.935, a sensitivity of 86.36%, and a specificity of 87.84%. Furthermore, Cyclin D1 dynamics were found to correlate significantly with NACT efficacy. A Cyclin D1 level exceeding 16.09 pg/mL at T1 predicted poor treatment response with 81.40% sensitivity and 45.05% specificity (AUC=0.652). When combined with parameters at T1, the model's predictive accuracy improved to 88.37% sensitivity and 56.76% specificity (AUC = 0.775). These findings highlight the potential of combining Cyclin D1 and MRI radiomics for a holistic BC assessment.

As is well known, Cyclin D1 is the core regulator of the G1/S phase transition of cell cycles. It forms a complex with CDK4/6 to phosphorylate RB protein and release E2F transcription factor, thus driving cells to enter the proliferation cycle [10]. Abnormally expressed Cyclin D1 in BC contributes to tumor progression through multiple mechanisms. Cell proliferation promotion: In vitro studies by Fang et al. demonstrated that Cyclin D1 overexpression shortens the G1 phase, accelerating tumor cell division and increasing tumor volume and invasiveness [11]. This aligns with our findings, confirming that elevated Cyclin D1 levels activate the malignant progression of tumor cells. Chemoresistance regulation: Cyclin D1 overexpression has been linked to ER-independent signaling pathways, potentially involving mechanisms such as MAPK/PI3K activation, which can contribute to impaired sensitivity to endocrine therapy [12]. Shi et al. found lower response rates to tamoxifen and shorter disease-free survival in BC patients with high Cyclin D1 expression [13]. However, emerging research highlights the multifactorial regulation of Cyclin D1, involving growth factor signaling, epigenetic modulation, and microenvironment hypoxia [14]. The complexity of these mechanisms suggests that Cyclin D1 may participate in BC occurrence and development through distinct pathways, underscoring the limitations of relying solely on Cyclin D1 as a biomarker for BC progression.

Consequently, this study attempts to establish a novel BC assessment model through Cyclin D1 and radiomics. Our findings demonstrate this integrated approach's superior diagnostic performance and NACT response prediction capability. We believe that this is due to the synergistic data integration of Cyclin D1 and imaging genomics, which makes up for the limitations of single-index detection to a great extent. For instance, although DCE-MRI parameters provide valuable vasopermeability information, they cannot distinguish proliferation-driven tumors from angiogenesis-dominated tumors [15]. Cyclin D1, as the core regulator of cell cycle progression, can directly reflect tumor proliferation activity [16], thereby complementing the molecular mechanistic insights that imaging modalities alone cannot provide. Meanwhile, as previously discussed, Cyclin D1 expression exhibits modulation by various biological factors. When combined with multiparametric MRI, which provides a quantitative assessment of intratumoral heterogeneity, this integrated approach significantly enhances tumor characterization and thus diagnostic accuracy.Our results demonstrate significant diagnostic improvement with the combined model (AUC = 0.935) compared to Cyclin D1 alone (AUC=0.830, 71.43% sensitivity, 82.43% specificity), suggestingthat the multimodal approach could effectively minimize false positive/false negative rates through complementary information integration to optimize diagnostic decision-making. Furthermore, our analysis revealed a significant positive correlation between early-phase Cyclin D1 reduction during NACT and subsequent tumor regression, supporting the clinical utility of serial Cyclin D1 quantification as a dynamic pharmacodynamic marker for treatment response monitoring. When integrated with parameters at T1, the model's predictive capacity was further enhanced, potentially by detecting early radiological alterations like post-chemotherapy vascular normalization or cellular apoptosis. It is worth noting that we used midtreatment (T1) biomarkers to establish the clinical utility of dynamic response assessment during NACT, which enables timely therapeutic adjustments, potentially improving patient outcomes through personalized treatment optimization.

The promising performance of the combined Cyclin D1 and MRI radiomics model suggests its potential clinical utility. Future studies should focus on validating this model in larger, multi-center cohorts to confirm generalizability. Integrating this model into clinical decision support systems could aid in: (1) Refining BC diagnosis, particularly for equivocal BI-RADS 4 lesions, potentially reducing unnecessary biopsies; (2) Early identification of patients likely to have a poor response to NACT, enabling timely treatment intensification or regimen switch.

However, while post-NACT test results were collected, incomplete prognostic follow-up limits our ability to utilize T3 data for prognostic risk evaluation currently. Additionally, although the case count determined by G-Power meets statistical power thresholds, broader validation through multi-center studies and larger cohorts remains necessary to ensure generalizability. furthermore, variability in MRI protocols (e.g., field strength, sequence parameters) and feature extraction methodologies (e.g., ROI segmentation, algorithmic selection) may introduce parameter inconsistencies, underscoring the need for standardized workflows. In the follow-up study, we need to correct the influence of potential confounding factors such as tumor staging and molecular typing on the expression of Cyclin D1 and parameters, so as to enhance the reliability of the conclusions.

## Conclusion

Cyclin D1 combined with MRI parameters can significantly enhance diagnostic accuracy for BC and improve predictions of NACT response. Cyclin D1 promotes tumor progression by regulating cell cycles and endocrine therapy resistance, while radiomics supplements complementary data on tumor heterogeneity (e.g., vascularization, cellular density). The integration of the two not only overcomes the limitations of single-index detection but also provides novel ideas for the formulation of individualized treatment strategies.

## Dodatak

### Acknowledgments

Authors thank all the participants from Sir Run Run shaw hospital for their contributions.

### Funding

The authors declare that no funds, grants, or other support were received during the preparation of this manuscript.

### Author contributions

X.Z. conceived and designed the study, YF.F. and YZ.S. wrote and revised the manuscript,Z.W. collected and analyzed the date, YF.F. and Z.W. visualisation the data and supervised the study, YF.F. and YZ.S. made equal contributions in this work as co-first authors. All authors read and approved the final submitted manuscript.

### Conflict of interest statement

All the authors declare that they have no conflict of interest in this work.
